# Association of surgical approach and prolonged opioid prescriptions in patients undergoing major pelvic cancer procedures

**DOI:** 10.1186/s12893-020-00879-5

**Published:** 2020-10-14

**Authors:** Marieke J. Krimphove, Xi Chen, Maya Marchese, David F. Friedlander, Adam C. Fields, Lina Roa, Daniel Pucheril, Adam S. Kibel, Nelya Melnitchouk, Richard D. Urman, Luis A. Kluth, Prokar Dasgupta, Quoc-Dien Trinh

**Affiliations:** 1 Division of Urological Surgery and Center for Surgery and Public Health, Brigham and Women’s Hospital, Harvard Medical School, 45 Francis St., ASB II–3, Boston, MA 02115 USA; 2 Department of Urology, University Hospital Frankfurt, Frankfurt, Germany; 3 Division of General and Gastrointestinal Surgery, Department of Surgery, Brigham and Women’s Hospital, Harvard Medical School, Boston, MA USA; 4 Program in Global Surgery and Social Change, Harvard Medical School, Boston, MA USA; 5 Department of Surgery and Center for Surgery and Public Health, Brigham and Women’s Hospital, Harvard Medical School, Boston, MA USA; 6 Department of Anesthesiology, Perioperative and Pain Medicine, Harvard Medical School, Brigham and Women’s Hospital, Boston, MA USA; 7 Department of Urology, King’s College London, Guy’s and St. Thomas’ Hospitals NHS Foundation Trust, Guy’s Hospital, London, UK

**Keywords:** Surgical approach, Minimally invasive surgery, Opioids

## Abstract

**Background:**

The rise in deaths attributed to opioid drugs has become a major public health problem in the United States and in the world. Minimally invasive surgery (MIS) is associated with a faster postoperative recovery and our aim was to investigate if the use of MIS was associated with lower odds of prolonged opioid prescriptions after major procedures.

**Methods:**

Retrospective study using the IBM Watson Health Marketscan® Commerical Claims and Encounters Database investigating opioid-naïve cancer patients aged 18–64 who underwent open versus MIS radical prostatectomy (RP), partial colectomy (PC) or hysterectomy (HYS) from 2012 to 2017. Propensity weighted logistic regression analyses were used to estimate the independent effect of surgical approach on prolonged opioid prescriptions, defined as prescriptions within 91–180 days of surgery.

**Results:**

Overall, 6838 patients underwent RP (MIS 85.5%), 4480 patients underwent PC (MIS 61.6%) and 1620 patients underwent HYS (MIS 41.8%). Approximately 70–80% of all patients had perioperative opioid prescriptions. In the weighted model, patients undergoing MIS were significantly less likely to have prolonged opioid prescriptions in all three surgery types (Odds Ratio [OR] 0.737, 95% Confidence Interval [CI] 0.595–0.914, *p* = 0.006; OR 0.728, 95% CI 0.600–0.882, *p* = 0.001; OR 0.655, 95% CI 0.466–0.920, *p* = 0.015, respectively).

**Conclusion:**

The use of the MIS was associated with lower odds of prolonged opioid prescription in all procedures examined. While additional studies such as clinical trials are needed for further confirmation, our findings need to be considered for patient counseling as postoperative differences between approaches do exist.

## Background

The United States is currently in the midst of a major opioid epidemic [1], with drug overdose supplanting motor vehicle collisions as the number one cause of accidental death [2]. The National Institute on Drug Abuse recently disclosed that more than 70,000 drug overdose deaths were registered in 2017 [2]. The rate of drug overdose deaths involving synthetic opioids other than methadone increased by 88% per year from 2013 to 2016 [2, 3]. Explanations for these trends include poor access to prevention, treatment, and recovery programs as well as more frequent opioid prescriptions [4, 5].

Major surgery may induce opioid use disorder in certain individuals as pain medication is prescribed on a regular basis after such procedures [6]. Expectations for no pain after surgery, as well as aggressive marketing to physicians from drug companies have led to increased use of opioids in the perioperative setting. For example, opioid prescriptions increased significantly from 2004 to 2014 in women undergoing hysterectomy for benign indications, even though less invasive techniques have emerged in recent years [7].

Minimally invasive surgery (MIS) is associated with certain perioperative benefits, such as decreased operative blood loss, shorter operation time, and length of stay, all of which have resulted in shorter recovery times following surgery [8, 9]. While prior research has focused on the perioperative period, little is known about intermediate term possible benefits MIS has on patients’ recovery.

Therefore, we sought to investigate differences in prolonged opioid prescription following MIS versus open surgery in common major cancer procedures. We hypothesized that the odds of prolonged opioid prescription is lower in patients undergoing MIS.

## Methods

### Data source

We queried the IBM Watson Health (formerly Truven Health Analytics) Marketscan® Commercial Claims and Encounters Database, which contains enrollment and healthcare (medical and drug) claims of millions of employees and their dependents who are covered annually under diverse health plans offered by medium or large sized firms. There are distinct data collected for inpatient, outpatient, emergency department, and outpatient prescription drug claims, all of which are linked by a unique patient identifier. From 2012 to 2017, the database contained de-identified claims for 1.87 million enrollees, 53% covered by self-insured employers and 47% covered by health plans.

The Marketscan® database only captures prescriptions filled at outpatient pharmacies and does not capture prescriptions filled within the hospital facility. Thus, inpatient opioid use could not be included in postoperative opioid use. Pharmacy claims data include the fill date, quantity supplied, and number of days supplied.

### Patient selection

The study cohort included patients aged 18–64 years of age, who underwent radical prostatectomy for prostate cancer, partial colectomy for colon cancer, or hysterectomy for uterine cancer between January 2012 and December 2017 (Additional file 1). Patients over the age of 64 were not included given their Medicare eligibility, a national health insurance program for elderly and disabled persons. The three procedures were selected based on both the public health significance with regard to overall volume and the contemporary prevalence of both open and MIS approaches for the procedure. If there were multiple claims for a given surgery, the earliest date was considered as the index surgical date. We analyzed individuals who were continuously enrolled for a period of at least 1 year prior to surgery up until 180 days after surgery. Patients with incomplete demographic data were excluded.

**Fig. 1:**
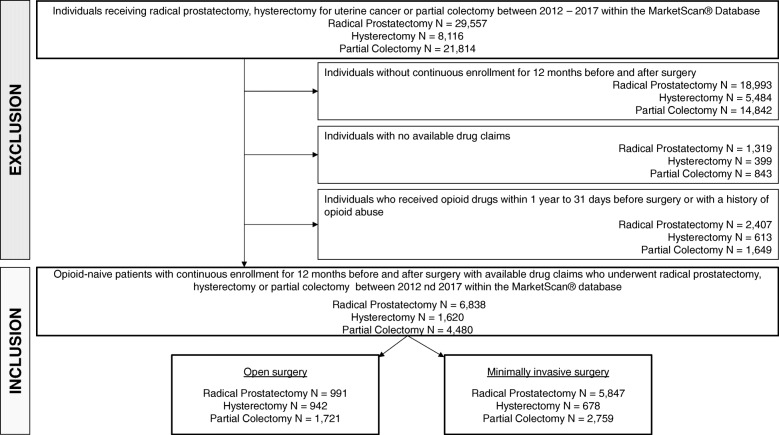
Cohort selection

We only included opioid naïve individuals, defined as patients with no opioid prescription within 1 year to 31 days before surgery and with no history of opioid use disorder (ICD-9 code 304.00–304.03; 305.5–305.53, and ICD-10 codes F 11.1x, F11.2x). Opioid prescriptions within 30 days before surgery were considered surgery-related and prescribed for postoperative pain management, as previously described (Fig. 1) [10, 11].

### Exposure of interest

The exposure of interest was surgical approach defined as open or MIS, including robotic surgery, of one of three major procedures: radical prostatectomy, partial colectomy, and hysterectomy.

### Outcomes

Consistent with prior conventions, our main outcome of interest was prolonged opioid prescription, defined as prescriptions filled within 91 to 180 days from surgery in patients who had also filled at least one prescription within the perioperative period, 30 days prior to 2 weeks after surgery [6, 10, 12]. This time period was chosen given that opioid use disorder is defined by the Centers for Disease Control and Prevention as opioid use for more than 3 months (91+ days).

### Covariates

Covariates included age (< 34, 35–44, 45–54, 55–64), sex, year of surgery, Elixhauser Comorbidity Index (0, 1, ≥2), US Census Region (Northeast, North Central, South, West, Unknown), Urban vs. Rural residence, colostomy (only for partial colectomy), low anterior resection (only for partial colectomy) and health plan type (less restrictive, more restrictive). Furthermore, we accounted for chronic opioid use risk factors for as described in literature [10, 12]. In brief, we examined if a patient had a claim with an ICD-9 code for depression, substance abuse other than opioids, and other mental health disorders (e.g. schizophrenia, mood disorders, etc.) (Additional file 2).

### Statistical analyses

Patient characteristics were compared by surgical approach (open vs. MIS) using t-tests for continuous variables or chi-square tests for categorical variables. We conducted an inverse probability of treatment weighted (IPTW) propensity score analysis to statistically pseudo-randomize the cohorts [13]. Therefore, we fit a logistic regression model and treatments were weighed by the inverse of their propensity score, while controls were weighted by the inverse of (1-propensity score). We controlled for all variables described above including the risk factors for opioid abuse, and year of diagnosis. An IPTW-weighted logistic regression was then used to assess the association between open versus MIS and prolonged opioid prescriptions.

All statistical analyses were performed using SAS 9.4 (SAS Institute Inc., SAS Campus Drive, Cary, North Carolina 27513, USA), a two-sided significance level was set at *p* < 0.05. Prior to performing this study we obtained an institutional review board waiver from Brigham and Women’s Hospital.

## Results

### Baseline characteristics

Table 1 summarizes the unweighted baseline characteristics of all patients who underwent one of the three procedures. In total, 6838 patients underwent radical prostatectomy, 4480 patients underwent partial colectomy, and 1620 patients underwent hysterectomy for uterine cancer. While radical prostatectomy and partial colectomy were performed in a MIS fashion for the majority of patients (85.5, and 61.6%, respectively), hysterectomy was mostly performed using the open approach (58.2%). All open procedures were significantly more often performed in southern states (all *p* < 0.05), open radical prostatectomy and partial colectomy were more often performed in rural areas (18.5% vs. 14.0 and 20.2% vs. 14.2%, respectively, both *p* < 0.05). Women undergoing MIS hysterectomy were significantly more often in the oldest age group (58.9 vs. 68.6%, *p* < 0.001).

**Table 1:** Baseline characteristics of opioid-naïve patients undergoing one of the following procedures: prostatectomy, partial colectomy, and hysterectomy between 2012 and 2017 within the Marketscan database

Procedure →	Prostatectomy *N* = 6838			Partial colectomy *N* = 4480			Hysterectomy *N* = 1620		
Co-variates↓	open 991 (14.5%)	MIS 5847 (85.5%)	*p*-value	open 1721 (38.4)	MIS 2759 (61.6)	*p*-value	open 942 (58.2)	MIS 678 (41.9)	*p*-value
**Age**	0.124			*0.028*			*< 0.001*		
18–34	0	1 (0.0)		33 (1.9)	41 (1.5)		11 (1.2)	10 (1.5)	
35–44	15 (1.5)	85 (1.5)		198 (11.5)	235 (8.5)		80 (8.5)	39 (5.8)	
45–54	220 (22.2)	1504 (25.7)		600 (34.9)	1052 (38.1)		296 (31.4)	164 (24.2)	
55–64	756 (76.3)	4257 (72.8)		890 (51.7)	1431 (51.8)		555 (58.9)	465 (68.6)	
**Gender**				0.718					
Male				865 (50.3)	1402 (50.8)				
female				856 (49.7)	1357 (49.2)				
**Elixhauser comorbidity**	0.462			*< 0.001*			0.252		
0	204 (20.6)	1268 (21.7)		184 (10.7)	344 (12.5)		111 (11.8)	100 (14.8)	
1	346 (34.9)	1929 (31.0)		369 (21.4)	658 (23.9)		206 (21.9)	139 (20.5)	
≥ 2	441 (44.5)	2650 (45.3)		1168 (67.9)	1757 (63.7)		625 (66.4)	439 (65.8)	
**Geographic region**	*< 0.001*			*< 0.001*			*< 0.001*		
Northeast	183 (18.5)	1225 (21.0)		324 (18.8)	563 (20.4)		218 (23.1)	170 (25.1)	
North central	193 (19.5)	1521 (26.0)		389 (22.6)	567 (22.6)		221 (23.5)	159 (23.5)	
South	459 (46.3)	2234 (38.2)		797 (46.3)	1202 (43.6)		363 (38.5)	175 (25.8)	
West	145 (14.6)	830 (14.2)		195 (11.3)	414 (15.0)		132 (14.0)	170 (25.1)	
Unknown	11 (1.1)	37 (0.6)		16 (0.9)	13 (0.4)		8 (0.9)	4 (0.6)	
**Residence**	*< 0.001*			*< 0.001*			0.456		
Rural	183 (18.5)	819 (14.0)		347 (20.2)	392 (14.2)		133 (14.1)	87 (12.8)	
Urban	808 (81.5)	5028 (86.0)		1374 (79.8)	2367 (85.8)		809 (85.9)	591 (87.2)	
**Health plan**	0.521			0.788			0.831		
Less restrictive	643 (64.9)	3855 (65.9)		1142 (66.4)	1820 (66.0)		626 (66.5)	454 (67.0)	
More restrictive	348 (35.1)	1992 (34.1)		579 (33.6)	939 (34.0)		316 (33.6)	224 (30.4)	
**Colostomy**				233 (13.5)	93 (3.4)	*< 0.001*			
**Low anterior resection**				360 (20.9)	764 (27.7)	*< 0.001*			
**Mental health disorders**									
**Depression**	51 (5.1)	319 (5.5)	0.691	103 (6.0)	193 (7.0)	0.185	62 (6.6)	57 (8.4)	0.165
**Substance abuse**	42 (4.2)	252 (4.3)	0.918	76 (4.4)	119 (4.3)	0.870	22 (2.3)	11 (1.6)	0.316
**Other**	30 (3.0)	226 (3.9)	0.199	75 (4.3)	107 (3.9)	0.429	41 (4.4)	28 (4.1)	0.827
**Opioid prescriptions**									
Perioperative	776 (78.3)	4680 (80.0)	0.208	1223 (71.1)	1980 (71.8)	0.613	761 (80.8)	536 (79.1)	0.390
Prolonged	65 (8.4)	262 (5.6)	*0.004*	223 (18.2)	240 (12.1)	*< 0.001*	109 (14.3)	50 (9.3)	*0.007*

*Abbreviation*: *MIS* minimally invasive surgery

### Unadjusted perioperative and prolonged opioid prescriptions

Approximately 70 to 80% of all patients had at least one perioperative opioid prescription, irrespective of surgery type and surgical approach (Table 1).

Prolonged use was most pronounced in patients who underwent open partial colectomy with 18.2%, followed by open hysterectomy (14.3%) and MIS partial colectomy (12.1%). In the unadjusted model, prolonged opioid prescriptions occurred significantly less often in individuals undergoing MIS for all three procedure groups. (all *p* < 0.05).

### Adjusted prolonged opioid prescriptions

In the IPTW logistic regression model, relative to patients undergoing conventional open surgery, patients undergoing MIS for all three procedures were less likely to have prolonged opioid prescriptions: radical prostatectomy, (Odds ratio [OR] 0.737, 95% Confidence Interval [CI] 0.595 to 0.914, *p* = 0.006), partial colectomy (OR 0.728, 95% CI 0.600 to 0.882, *p* = 0.001), and hysterectomy (OR 0.655 95% CI 0.466 to 0.920, *p* = 0.015) (Table 2).

**Table 2:** IPTW-weighted logistic regression predicting prolonged opioid prescription in opioid-naive patients undergoing radical prostatectomy, partial colectomy or hysterectomy

	Radical prostatectomy			Partial colectomy			Hysterectomy		
	OR	95%-CI	*p*-value	OR	95%-CI	*p*-value	OR	95%-CI	*p*-value
Open	Ref.			Ref.			Ref.		
MIS	0.737	0.595–0.914	0.006	0.728	0.600–0.882	0.001	0.655	0.466–0.920	0.015

Weighted for: Year, Age, Sex, Elixhauser Comorbidity, Geographic region, Residence, Health Plan, Mental Health Disorders, Colostomy, Low Anterior Resection

*Abbreviations*: *MIS* minimally invasive surgery, *OR* Odds ratio, *95%-CI* 95% Confidence Interval, *Ref*. Reference

## Discussion

Against the background of increasing death rates from opioid use, opioid prescriptions following major surgical procedures are perceived as possible precipitating factors of opioid misuse and abuse [14]. Patients undergoing surgical procedures often receive opioid prescriptions with unclear instructions and expectations. Moreover, prescriptions are often renewed when faced with patient complaints, with little investigation into the cause of the symptoms and the appropriateness of the opioid medication for the problem [15]. Our study sought to investigate differences in prolonged opioid prescriptions following open and MIS approaches for radical prostatectomy, partial colectomy and hysterectomy adding a new perspective to a growing body of work, investigating opioid prescription pattern [11, 16].

Prolonged prescription use was less common after minimally invasive radical prostatectomy partial colectomy, and hysterectomy relative to the respective open approaches. To our knowledge, this is the first study to demonstrate the intermediate-term benefit of MIS with regard to opioid use. Though novel, our findings are consistent with previous findings of decreased perioperative analgesia requirements, and quicker recuperation afforded by MIS [17, 18]. Therefore, the increased perioperative costs of MIS, including robotic surgery may be offset by long-term advantages such as quicker return to work and decreased opioid utilization [19, 20].

From a practical perspective, our findings are important to consider when more than half of patients who receive 90 days of continuous opioid therapy will remain on opioids years later [21]. Similarly, in opioid naïve patients, those with opioid prescriptions within 7 days of surgery are 44% more likely to become long term opioid users [22]. Further, data from trauma surgery has demonstrated that up to 8.8% of patients continue using opioids 6 months after surgery [23–25]. While the relationship between early opioid use and long-term addiction is still poorly understood, one possible explanation may be early desensitization of opioid receptors after acute opioid administration [26] requiring more opioids in order to achieve the same level of pain relief (i.e. acute opioid tolerance and hyperalgesia). This in turn leads to adaptive tolerance of opioid receptors, feeding the downward spiral to opioid addiction. Additionally, there are other neurobiological [27], psychological [28, 29], and personal factors [30] involved in the complex interaction that results in opioid use disorder. Nonetheless, the probability of long-term opioid use increases most sharply in the first days of therapy [31]; thus, to prevent long-term opioid use it is crucial to transition to less addictive analgesic alternatives early in the post-operative course.

Of note, we found that 70 to 80% of all patients – irrespective of site and type of surgical approach – receive perioperative opioid prescriptions. Opioids are effective drugs in treating acute pain, nevertheless opioids are highly addictive and even short-term use can result in unintentional prolonged use with a risk of abuse and dependence [32]. Similar results were seen in patients who undergo low-risk surgery. Wunsch et al. found that within 7 days from surgery, 80% filled a prescription for any opioid – with an increasing trend over time [33]. Postoperative pain is common in patients undergoing major surgical procedures and pain management is most crucial in terms of preventing chronic pain with associated decrease of quality of life [34]. In 2016, a Clinical Practice Guideline from the American Pain Society, the American Society of Regional Anesthesia and Pain Medicine, and the American Society of Anesthesiologists’ Committee on Regional Anesthesia advised that safe and effective postoperative pain management should be tailored to the individual and the surgical procedure involved [35]. For example, following radical prostatectomy, many advocate the primary use of non-opioid analgesics and/or regional analgesic techniques with opioids utilized only when necessary [36]. However, despite efforts to curtail opioid over-utilization, postsurgical pain management remains poorly understood. Patel et al. found that in patients undergoing radical prostatectomy, 77% of opioids prescribed were unused, with 84% of patients using less than half of their prescription [37]. It is possible, that physicians routinely prescribe opioid drugs following surgery, without taking into account the actual patient’s need.

Our study has several limitations. First, the retrospective design leaves room for unmeasured confounding. For example, we cannot account for surgical complexity – cases selected for the open approach may be more technically complex and at risk for complications than MIS cases. Second, we looked at prescription rates following surgery which may be different from the patient’s actual consumption of opioid drugs. More than 50% of patients are using less than half of the prescribed opioids [37, 38]. However, many opioid drugs are in uncontrolled circulation lacking medical monitoring. In theory, these overprescriptions may be diverted to other individuals in the setting of illegal resale. Regardless, Howard et al. found that prolonged prescription is correlated with increased consumption [39]. Third, we were not able to account for the effect of cancer stage on opioid prescriptions as this information was not available in the database. Patients presenting with more advanced disease at presentation may be preferentially managed with open surgery. Such cases are more at risk for progression and metastasis, which may require opioid use for palliative pain control. What’s more, the database does not contain information on the incision made during open surgery which might impact postoperative pain (e.g. pfannenstiel vs. midline incision). Fourth, data mostly come from large employers and medium and small firms may be underrepresented [40]. Moreover, this analysis excludes under- and un-insured individuals, and therefore is not representative of the US population as a whole. Fifth, we did not account for different postoperative management pathways including the use of enhanced recovery after surgery that may explain differences between prolonged opioid prescriptions rather than the surgical approach itself [41].

## Conclusion

The use of the MIS was associated with lower odds of prolonged opioid prescription in all procedures examined. While additional studies such as clinical trials are needed for further confirmation, our findings need to be considered for patient counseling as postoperative differences between approaches do exist.

## Supplementary information

**Additional file 1.** ICD-9, ICD-10 and CPT codes for disease states and procedures

**Additional file 2 .** ICD-9 and ICD-10 codes for risk factors of opioid use disorder

## Data Availability

The data of this study is from IBM Marketscan® and we do not have the authority to share.

## Abbreviations

CI: Confidence interval
HYS: Hysterectomy
IPTW: Inverse probability of treatment weighted
MIS: Minimally invasive surgery
OR: Odds ratio
PC: Partial colectomy
RP: Radical prostatectomy
