# Supplementary material for: Association of surgical approach and prolonged opioid prescriptions in patients undergoing major pelvic cancer procedures
Source: BMC Surg. 2020 Oct 14;20:235. doi: 10.1186/s12893-020-00879-5 (PMC7557098; doi:10.1186/s12893-020-00879-5)
Supplement: Supplementary file 1 — Additional file 1. ICD-9, ICD-10 and CPT codes for disease states and procedures [file 12893_2020_879_MOESM1_ESM.docx]

**Additional file 1**

ICD-9, ICD-10 and CPT code for disease states and procedures

| **Category** | **ICD 9 – DX** | **ICD 10 – DX** | **Procedure** | **ICD 9 – Proc** | **ICD 10 – Proc** | **CPT** |
| --- | --- | --- | --- | --- | --- | --- |
| **Prostate Cancer** | 185 | C61 | **Open Radical Prostatectomy** | 60.5 | 0VT00ZZ, 0VT07ZZ | 55840, 55842, 55845 |
|  |  |  | **Laparoscopic Radical Prostatectomy** | N/A | 0VT04ZZ, 0VT08ZZ | 55866 |
|  |  |  | **Robotic Assisted Laparoscopic Prostatectomy** | Above + 17.4, 17.41, 17.42, 17.43, 17.44, 17.45, 17.49 | Above + 8E0W4CZ, 8E0W7CZ, 8E0WXCZ, 8E0W0CZ, 8E0W3CZ, 8E0W8CZ | Above + S2900 (HCPCS) |
| **Cervical and Endometrial/Uterine Cancer** | 179, (180, 180.0, 180.1, 180.8, 180.9) (182, 182.0, 182.1, 182.8) | C55, C53, C53.0, C53.1, C53.8, C53.9, C54, C54.0, C54.1, C54.2, C54.3, C54.8, C54.9 | **Open Total Abdominal Hysterectomy / Radical Abdominal Hysterectomy** | 68.4, 68.49, 68.6, 68.69 | 0UT90ZZ + 0UTC0ZZ | 58150, 58152, 58200, 58210 |
|  |  |  | **Laparoscopic Total Abdominal/ Radical Abdominal Hysterectomy** | 68.41, 68.61 | 0UT94ZZ + 0UTC4ZZ | 58548, 58570, 58571, 58572, 58573, 58575, 58578 + any open, 58579 + any open |
|  |  |  | **Robotic Assisted Total Abdoinal Hysterectom/Radical Abdominal Hysterectomy** | Above + 17.4, 17.41, 17.42, 17.43, 17.44, 17.45, 17.49 | Above + 8E0W4CZ, 8E0W7CZ, 8E0WXCZ, 8E0W0CZ, 8E0W3CZ, 8E0W8CZ | Above + S2900 (HCPCS) |
|  |  |  | **Open Myomectomy** | 68.29 | 0U590ZZ, 0U597ZZ, 0UB90ZZ, 0UB97ZZ | 58140  58146 |
|  |  |  | **Laparoscopic Myomectomy** | N/A | 0U593ZZ, 0U594ZZ, 0U598ZZ, 0UB93ZZ, 0UB94ZZ, 0UB98ZZ | 58545  58546 |
|  |  |  | **Robotic Assisted Myomectomy** | Above + 17.4, 17.41, 17.42, 17.43, 17.44, 17.45, 17.49 | Above + 8E0W4CZ, 8E0W7CZ, 8E0WXCZ, 8E0W0CZ, 8E0W3CZ, 8E0W8CZ | Above + S2900 (HCPCS) |
| **Colon Cancer** | 153, 153.0, 153.1, 153.2, 153.3, 153.4, 153.5, 153.6, 153.7, 153.8, 153.9 | C18, C18.0, C18.1, C18.2, C18.3, C18.4, C18.5, C18.6, C18.7, C18.8, C18.9 | **Open Partial Colectomy** | 45.41, 45.71, 45.72, 45.73, 45.74, 45.75, 45.76, 45.79, 45.7 | 0DTH0ZZ, 0DTH7ZZ, 0DTK0ZZ, 0DTK7ZZ, 0DTF0ZZ, 0DTF7ZZ,  0DTL0ZZ,  0DTL7ZZ,  0DTM0ZZ,  0DTM7ZZ,  0DTG0ZZ,  0DTG7ZZ, 0DTN0ZZ,  0DTN7ZZ,  0DBE0ZZ, 0DBE7ZZ, 0DBH0ZZ,  0DBH7ZZ, 0DBK0ZZ,  0DBK7ZZ, 0DBF0ZZ, 0DBF7ZZ, 0DBL0ZZ, 0DBL7ZZ, 0DBM0ZZ, 0DBM7ZZ, 0DBG0ZZ,  0DBG7ZZ, 0DBN0ZZ,  0DBN7ZZ | 44140, 44141, 44143, 44144, 44145, 44146, 44147, 44160 |
|  |  |  | **Laparoscopic Partial Colectomy** | 17.3, 17.31, 17.32, 17.33, 17.34, 17.35, 17.36, 17.39 | 0DTH4ZZ, 0DTH8ZZ, 0DTK4ZZ, 0DTK8ZZ,  0DTF4ZZ, 0DTF8ZZ,  0DTL4ZZ,  0DTL8ZZ,  0DTLFZZ,  0DTM4ZZ, 0DTM8ZZ,  0DTMFZZ,  0DTG4ZZ,  0DTG8ZZ,  0DTGFZZ, 0DTN4ZZ,  0DTN8ZZ,  0DTNFZZ, 0DBE3ZZ,  0DBE4ZZ,  0DBE8ZZ, 0DBH3ZZ,  0DBH4ZZ,  0DBH8ZZ, 0DBK3ZZ,  0DBK4ZZ,  0DBK8ZZ, 0DBF3ZZ,  0DBF4ZZ,  0DBF8ZZ, 0DBL3ZZ,  0DBL4ZZ,  0DBL8ZZ,  0DBLFZZ, 0DBM3ZZ,  0DBM4ZZ,  0DBM8ZZ,  0DBMFZZ, 0DBG3ZZ,  0DBG4ZZ,  0DBG8ZZ,  0DBGFZZ,  0DBN3ZZ,  0DBN4ZZ,  0DBN8ZZ,  0DBNFZZ | 44204, 44205, 44206, 44207, 44208, |
|  |  |  | **Robotic Assisted Partial Colectomy** | Above + 17.4, 17.41, 17.42, 17.43, 17.44, 17.45, 17.49 | Above + 8E0W4CZ, 8E0W7CZ, 8E0WXCZ, 8E0W0CZ, 8E0W3CZ, 8E0W8CZ | Above + S2900 (HCPCS) |
