# Supplementary material for: Association of surgical approach and prolonged opioid prescriptions in patients undergoing major pelvic cancer procedures
Source: BMC Surg. 2020 Oct 14;20:235. doi: 10.1186/s12893-020-00879-5 (PMC7557098; doi:10.1186/s12893-020-00879-5)
Supplement: Supplementary file 2 — Additional file 2 . ICD-9 and ICD-10 codes for risk factors of opioid use disorder [file 12893_2020_879_MOESM2_ESM.docx]

**Additional file 2**

ICD-9 and ICD-10 codes for risk factors of opioid use disorder

| **Risk Factors** | | **ICD-9** | **ICD-10** |
| --- | --- | --- | --- |
| Depression | | 296.2, 296.3, 296.5, 300.4, 309.0 309.1, 311.xx | F20.4, F31.3, F31.4, F31.5, F32.x, F33.x, F34.1, F41.2, F43.2 |
| Substance Abuse | | 291.xx, 292.xx, 303.xx, 304.xx, 305.xx, V65.42, 265.2, 357.5, 425.5, 535.3x, 571.xx, 980.xx, V113 | F10.x, F11.x, F12.x, F13.x, F14.x, F15.x, F16.x, F17.x, F18.x, F19.x, Z71.5, Z72.2, E52, G62.1, I42.6, K29.2, K70.x, T51.x, Z50.2, Z71.4, Z72.1 |
| Other Mental Health Disorders  (any code + no depression) | Schizophrenia | 295.xx | F20.x |
|  | Mood Disorder/Bipolar Disorder | 296.xx | F30.x, F31.x, F34.x, F39.x |
|  | Anxiety | 300.xx | F40.x, F41.x, F42.x, F44.x, F45.x, F48.x |
|  | Personality Disorder | 301.xx | F60.x, F68.x, F69.x |
